# Pathway complexity in the self-assembly of a zinc chlorin model system of natural bacteriochlorophyll J-aggregates†
†Electronic supplementary information (ESI) available: Materials and methods, supplementary figures and a table. See DOI: 10.1039/c7sc03725b


**DOI:** 10.1039/c7sc03725b

**Published:** 2018-02-14

**Authors:** Soichiro Ogi, Charlotte Grzeszkiewicz, Frank Würthner

**Affiliations:** a Universität Würzburg , Institut für Organische Chemie , Am Hubland , 97074 Würzburg , Germany . Email: wuerthner@uni-wuerzburg.de; b Universität Würzburg , Center for Nanosystems Chemistry (CNC) , Bavarian Polymer Institute (BPI) , Theodor-Boveri-Weg , 97074 Würzurg , Germany

## Abstract

Whilst bacteriochlorophyll *c*, *d*, and *e* dyes self-assemble into the most efficient light harvesting J-aggregate systems found in nature, their supramolecular packing arrangements are still a matter of debate and a significant number of models have been suggested for their local and long-range ordering. Here we reveal for a synthetic model system based on a zinc chlorin (ZnChl) dye an intriguing interplay of two competing aggregation pathways by kinetic and thermodynamic studies in MeOH/water solvent mixtures: the formation of kinetically controlled off-pathway nanoparticles consisting of excitonically coupled J-dimers *versus* the formation of thermodynamically more stable one-dimensional helical fibers consisting of J-coupled extended aggregates. The higher order of the latter is evidenced by atomic force microscopy and a more narrow absorption spectrum of the J-aggregates. Based on a recently developed thermodynamic model that combines the cooperative *K*_2_–*K* growth model with a competing dimerization model, an energy landscape could be derived that describes the pathway complexity of this biomimetic system. Our studies reveal that the kinetic stability of the off-pathway nanoparticles increases with increasing concentration of ZnChl or water content in a MeOH/water solvent mixture. For a water content >90% deeply trapped off-pathway nanoparticle products are formed that do not transform anymore to the more ordered thermodynamic product within reasonable time scales. Based on these observations, we hypothesize that out-of-equilibrium aggregate structures of natural BChl dyes may also exist in the natural chlorosomes of green bacteria.

## Abstract 2

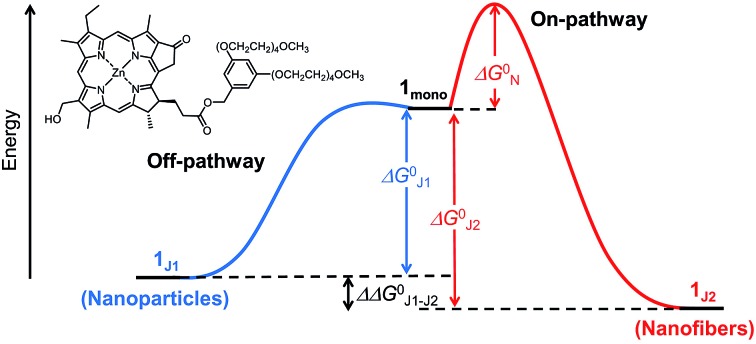
Self-assembly studies of a model compound of bacteriochlorophyll revealed the formation of nanoparticles as off-pathway and nanofibers as on-pathway products.

## Introduction

**Fig. 1:**
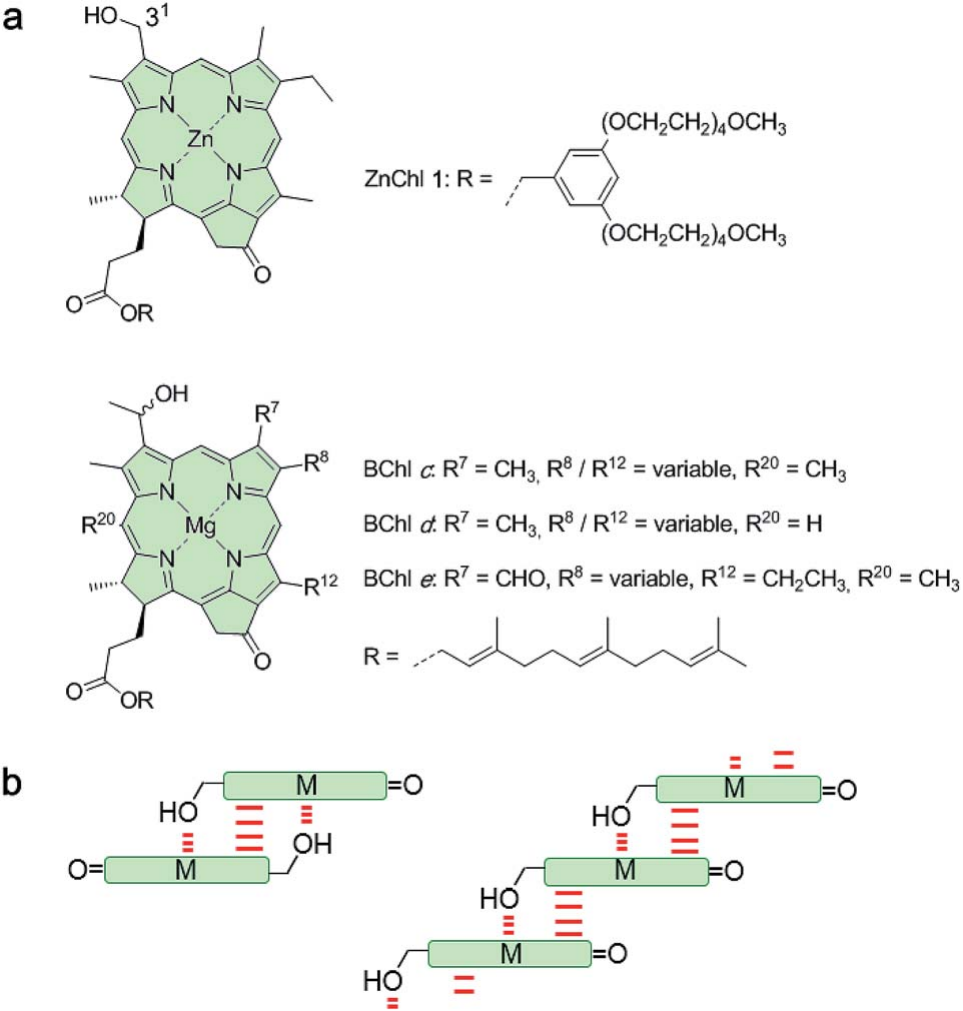
(a) Chemical structures of the semi-synthetic model compound ZnChl **1** and natural BChl *c*, *d*, and *e* molecules. (b) Schematic illustration of the antiparallel (left) and the parallel (right) one-dimensional stacking arrangement proposed for BChl *c*, *d*, and *e* aggregates.

Despite the vast amount of research accomplished in the last few decades on self-assembly,^1^ there is still an apparent gap between the mechanistic understanding of self-assembly processes of synthetic systems so far achieved^2^ and those that occur in nature, *e.g.* the formation of tobacco mosaic virus,^3^ amyloid fibers^4^ or biological bilayer membranes.^5^ A prime example where chemistry and biology meet is that of chlorosomes.^6^ These are light-harvesting antenna systems of green bacteria composed of self-assembled metallochlorin dyes, in particular bacteriochlorophylls (BChls) *c*, *d*, and *e* that possess a chlorophyll scaffold bearing a hydroxyl group at the 3^1^-position and a metal ion at the center (Fig. 1a).6c,^7^ The hydroxyl group is responsible for the slipped π-stacking arrangement into J-aggregates upon self-assembly of these natural BChl dyes driven by coordinative bonding to the metal ion of the neighbouring tetrapyrrole scaffold.^8^ Whilst this particular interaction is undisputed, the macroscopic morphology (sheets, tubes, and onions) and specific molecular arrangement of BChl dyes (parallel and antiparallel stacking arrangement, see Fig. 1b) in natural chlorosomal self-assemblies have been controversially discussed in the literature for decades.^9^ However, so far little attention has been paid to the self-assembly mechanism^10^ and the possibility of competing self-assembly pathways for natural BChl dyes^7^,^8^ or their semi-synthetic metallochlorin model compounds.^11^ The reason might be that it is very challenging to simulate the conditions that occur in natural environments and that many of the mathematical models for the characterization of self-assembly processes became available only in recent years.^2^

Motivated by significant recent progress in the mechanistic understanding of aggregation processes,^2^ we here elucidate the self-assembly mechanism of a semi-synthetic zinc chlorin model compound (ZnChl **1**)11*d* (Fig. 1a) by kinetic and thermodynamic studies. This model compound is, like natural BChls, inherently chiral and contains a hydroxyl group at the 3^1^-position and a central metal ion. We have previously reported that the hydroxyl group confines the self-assembly of ZnChl **1** into one-dimensional aggregates through the formation of coordinative bonds with the central zinc ions of neighbour molecules and directs the further growth of the one-dimensional stacks into tubes by hydrogen bonding to carbonyl groups of other stacks.8b,11d

We are aware that the self-assembly pathways given in natural chlorosomes are more complex than those of semi-synthetic model systems due to the presence of different BChl dyes, including variations in diastereomers,7b,11a,^12^,^13^ and additional possibilities of growth and the embedding of the natural dyes within a lipid monolayer membrane. However, due to the structural similarity of the model compound ZnChl **1** to natural BChls our present mechanistic exploration will also shed light on the natural counterparts, in particular with regard to the first level of hierarchical growth, *i.e.* the formation of slipped J-aggregate π-stacks. Here we report that ZnChl **1** self-assembles in methanol (MeOH)/water solvent mixtures into kinetically trapped nanoparticle aggregates that are structurally and spectroscopically distinct from the thermodynamically equilibrated J-aggregate helical fibers. At high concentrations of ZnChl **1** and with a high water content in the solvent mixtures the out-of-equilibrium nanoparticles prevail for a very long time as a deeply trapped state. Thermodynamic analysis performed for the higher methanol content, where equilibration is fast, suggests that the nanoparticle aggregates consisting of antiparallel ZnChl dimers are an off-pathway product and one-dimensional fibril aggregates with parallel stacks are a thermodynamic product.

## Results and discussion

**Fig. 2:**
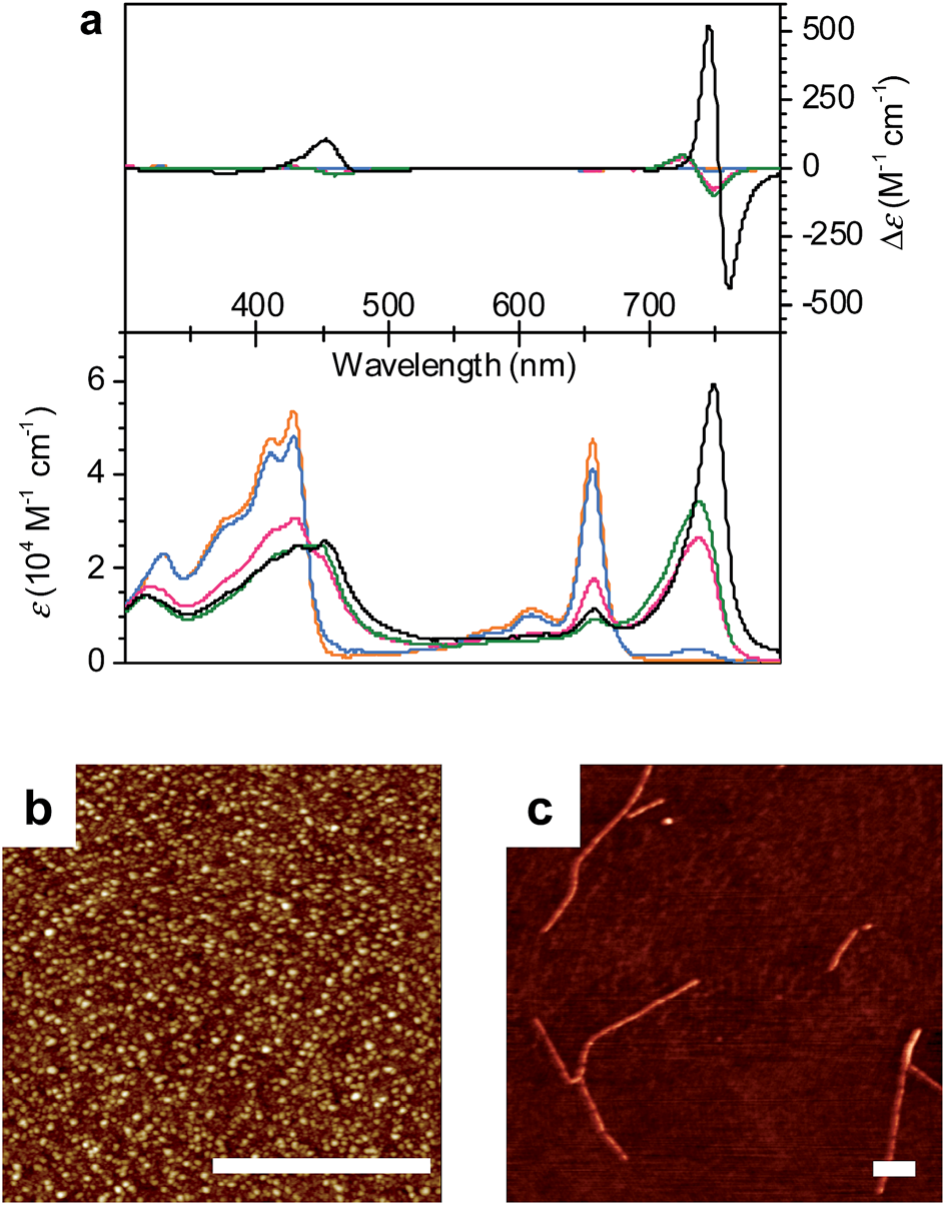
(a) Solvent-dependent CD and UV/vis absorption spectra of freshly prepared solutions of **1** in MeOH/water solvent mixtures with volume ratios of 50 : 50 (orange lines), 40 : 60 (blue lines), 30 : 70 (pink lines), and 20 : 80 (green lines) at a concentration of 1 × 10^–5^ M and a temperature of 293 K. The black lines indicate CD and UV/vis absorption spectra of ZnChl **1** in a 30 : 70 MeOH/water solvent mixture observed after 24 h at 293 K (*c*_T_ = 1 × 10^–5^ M). (b and c) AFM height images of (b) **1_J1_** spin-coated onto a silicon wafer from a freshly prepared solution of ZnChl **1** in 30 : 70 MeOH/water and (c) **1_J2_** spin-coated onto a silicon wafer from this solution stored in the absence of light for a few weeks. The scale bar is 500 nm. The *z* scales are (b) 5 nm and (c) 4.5 nm.

The self-assembly behaviour of ZnChl **1** was investigated by solvent-dependent UV/vis absorption and circular dichroism (CD) spectroscopy. For the sample preparation, ZnChl **1** was dissolved in methanol and then deionized water was added to the monomer solution in methanol until the water content reached 50–80 vol% with a total concentration (*c*_T_) of 1 × 10^–5^ M. The absorption spectrum of **1** in a 50 : 50 MeOH/water mixture showed a molecularly dissolved state (**1_mono_**) with the characteristic Soret band at 428 nm and the *Q*_y_ band at 656 nm (Fig. 2a, orange line).11*d* Upon increasing the volume ratio of water in the MeOH/water mixture from 50 : 50 to 20 : 80, the monomer *Q*_y_ band with a full width at half maximum (FWHM) of 450 cm^–1^ decreased and a new broad *Q*_y_ band (FWHM = 850 cm^–1^) appeared at 738 nm (Fig. 2a, green line). The bathochromic shift of the *Q*_y_ absorption maximum by 82 nm in the 20 : 80 MeOH/water mixture was accompanied by the appearance of bisignate CD signals with positive and negative maxima at around 725 and 749 nm, respectively (Fig. 2a, green line). These spectral features are indicative of the transition from **1_mono_** to a J-aggregate state (denoted as **1_J1_**) with chiral excitonic coupling^14^ of the *Q*_y_ transition dipole moments.

Interestingly, the absorption and CD spectra of **1_J1_** further changed over time with a higher water content of 30 : 70 in the MeOH/water mixture at a concentration of 1 × 10^–5^ M. Thus, the *Q*_y_ absorption band of **1_J1_** at 738 nm was bathochromically shifted to 749 nm with a pronounced increase of intensity and a narrowing of the band (FWHM = 510 cm^–1^) after 24 h (Fig. 2a, black line). Additionally, a strongly intensified CD couplet was observed at 745 and 760 nm as positive and negative maxima, respectively. The time-dependent spectral changes clearly indicate that **1_J1_** is a kinetic self-assembly product, which is transformed into a thermodynamically equilibrated J-aggregate state (denoted as **1_J2_**). This transformation from **1_J1_** to **1_J2_** is characterized by the anisotropy factor *g* (defined as Δ*ε*/*ε*) at 745 nm that increased from –2.7 × 10^–3^ to 9.4 × 10^–3^. The strong increase of the *g* value during the transformation from **1_1J_** to **1_J2_** suggests a different spatial arrangement of the dyes and a more extended and highly ordered aggregate structure in the thermodynamically equilibrated **1_J2_** state. Indeed, atomic force microscopy (AFM) images of a freshly prepared solution of **1_J1_** in the 30 : 70 MeOH/water mixture showed self-assembled nanoparticles (Fig. 2b), while fibrous nanostructures with a unimolecular width were observed for the **1_J2_** sample (Fig. 2c and S1 in the ESI†). Taking into account the structural proposals discussed in the literature for aggregates of similar metallochlorins based on UV/vis and CD spectroscopic studies,^11^ as well as scanning tunneling microscopy studies,^15^ it is reasonable to relate the **1_J1_** state to closely π-stacked antiparallel dimers (small CD effects are due to very small rotational displacements of the transition dipole moments) whose further aggregation into nanoparticles lacks high order. In contrast, the **1_J2_** state with its elongated helical fibrous structure, large CD amplitude and much sharper UV/vis absorption band is in accordance with the widely favoured parallel π-stack models (compare Fig. 1b) for natural BChl self-assemblies in the chlorosome,^9^ where the one-dimensional metallo-supramolecular chain is directed into a helical arrangement by the inherent chirality of the chlorin scaffold.

**Fig. 3:**
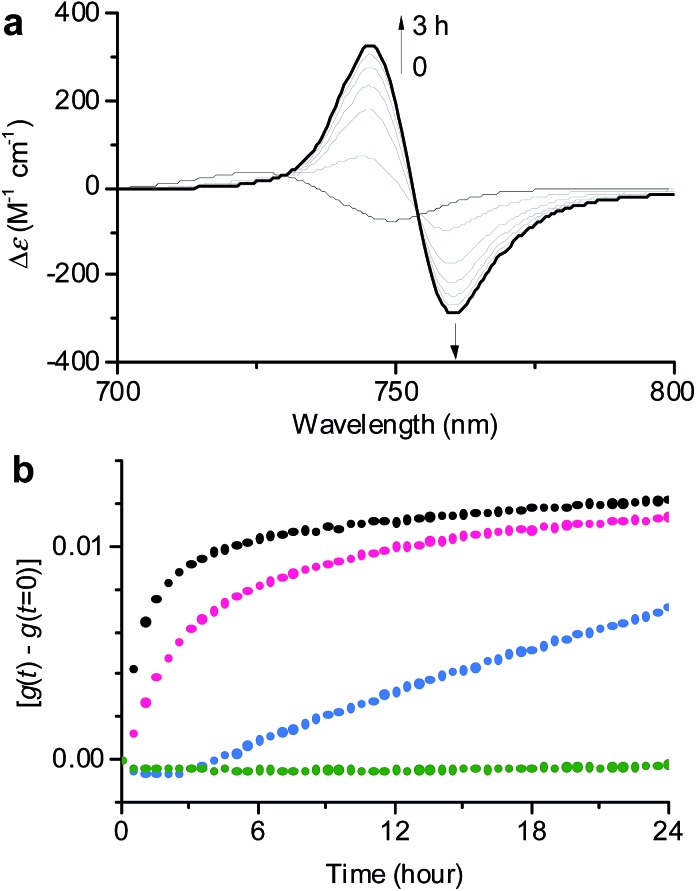
(a) Time-dependent CD spectral changes showing the transformation from **1_J1_** to **1_J2_** in a 30 : 70 MeOH/water solvent mixture at *c*_T_ = 1 × 10^–5^ M and 293 K. The arrows indicate spectral changes with time increasing from 0 to 3 h. (b) Time course of the *g* value changes at 745 nm showing the transformation from **1_J1_** into **1_J2_** in various MeOH/water mixtures of 30 : 70 (black), 20 : 80 (pink), 10 : 90 (blue), and 1 : 99 (green) at a concentration of 1 × 10^–5^ M and a temperature of 293 K. The rates for the transformation from **1_J1_** to **1_J2_** are shown in Table S1 in the ESI.†

To gain insight into the transformation mechanism from **1_J1_** into **1_J2_**, the aggregation kinetics at a temperature of 293 K was evaluated by monitoring the time-dependent changes of the UV/vis and CD spectra (*ε* and *g* values, respectively) at different total concentrations of ZnChl **1** in a 30 : 70 MeOH/water mixture (Fig. 3 and S2 in the ESI†). An important finding of this kinetic study is that the initial transformation rate decreases with an increased total concentration of **1** (Table S1 in the ESI†). This concentration dependence of the kinetics resembles those previously reported for porphyrin and perylene bisimide aggregate systems, in which the nanoparticles are off-pathway intermediates with regard to the thermodynamically stable supramolecular polymer,^16^ and is opposed to a system in which the nanoparticles are on-pathway aggregates that further grow into nanosheets.^17^ Thus, our concentration-dependent data support the conclusion that the kinetically formed **1_J1_** nanoparticle state is an off-pathway non-equilibrium aggregate,^18^ which is not used as a building block for the formation of thermodynamically stable fibrous J-aggregate **1_J2_**.

In general, the thermodynamic stability of π-conjugated dye aggregates increases with a higher water content due to hydrophobic solvation,^19^ which in our case leads to a deeper kinetic trapping for the **1_J1_** species. This issue has been addressed by time-dependent CD spectroscopic measurements of **1** in various MeOH/water mixtures from 30 : 70 up to 1 : 99 at a concentration of 1 × 10^–5^ M and 293 K (Fig. 3). Indeed, at this concentration the kinetics for the transformation process (d(*g*)/d*t*) from **1_J1_** into **1_J2_** decreased from 8.6 × 10^–3^ h^–1^ to 2.5 × 10^–3^ h^–1^ with increasing water content from 70% to 80% (Fig. 3b, black and pink dots). A freshly prepared solution of **1** in the 10 : 90 MeOH/water mixture showed the absorption spectrum of **1_J1_** (Fig. S3a in the ESI†), which was kinetically trapped for 3 h and transformed into **1_J2_** with a rate (d(*g*)/d*t*) of only 0.4 × 10^–3^ h^–1^ (Fig. 3b, blue dots). For the 1 : 99 MeOH/water mixture no noticeable transformation was observed on the experimental timescale (Fig. 3b, green dots), clearly indicating that the off-pathway **1_J1_** has high kinetic stability in water (Fig. S3b in the ESI†).^19^ AFM images of a freshly prepared sample of **1** in the 1 : 99 MeOH/water solvent mixture showed the presence of nanoparticles, which remained unchanged for several days (Fig. S4 in the ESI†). Thus, the **1_J1_** aggregate represents a very stable dispersion of the metallochlorin that can be stored for a long period of time. This observation now explains why the preparation of the highly defined nanotubular aggregate of ZnChl **1** in water-rich solutions required a very long time of up to several months.8*b*

**Fig. 4:**
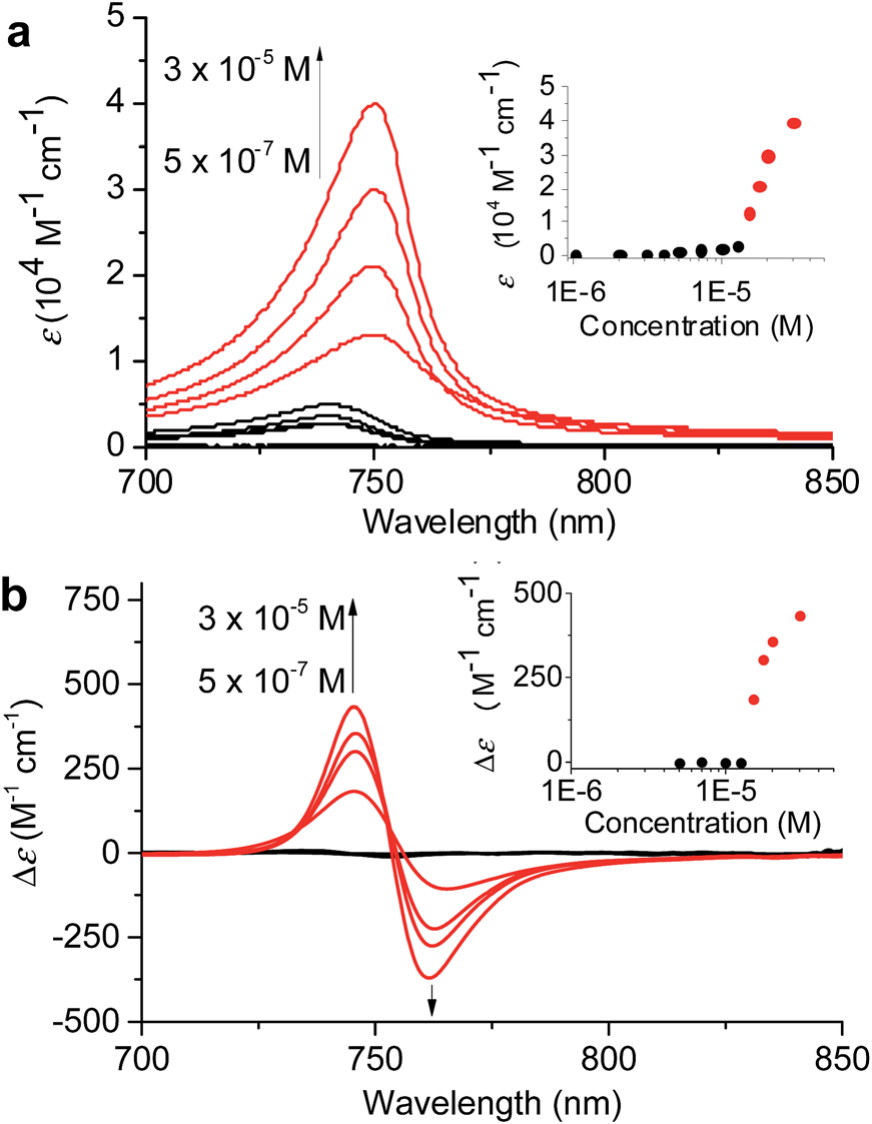
(a) Concentration-dependent UV/vis absorption and (b) CD spectral changes showing the formation of **1_J1_** and **1_J2_** in a 40 : 60 MeOH/water solvent mixture at 293 K. The arrows indicate spectral changes with increasing concentration from 5 × 10^–7^ to 3 × 10^–5^ M^–1^. Insets: plots of *ε* values at 750 nm and Δ*ε* values at 746 nm as a function of concentration.

To verify the existence of two competing aggregation pathways, we have further investigated the concentration-dependent self-assembly behaviour of ZnChl **1** at 293 K based on thermodynamic analysis. In this study, a solvent mixture of 40 : 60 MeOH/water was chosen because in this solvent mixture **1** is fully dissolved monomerically at low concentrations and the equilibrium between **1_mono_**, **1_J1_**, and **1_J2_** is rapidly reached with a higher MeOH content even with higher concentrations. The concentration-dependent absorption spectral changes for fully equilibrated solutions confirmed the formation of **1_J1_** as characterized by the appearance of a weak *Q*_y_ absorption band at 740 nm in the lower concentration (<7 × 10^–6^ M) range (Fig. 4a, black lines and black dots in the inset). In this concentration range the prevailing species are still monomers, which however start to self-assemble into dimeric species upon increasing the concentration. At concentrations above 1.2 × 10^–5^ M the *Q*_y_ absorption band with its maximum now at 750 nm strongly intensified and the positive and negative CD signals at 745 and 761 nm intensified in the same manner (Fig. 4, red lines and red dots in the inset).

The non-sigmoidal curves shown in the insets of Fig. 4 are indicative of a cooperative growth of monomeric species into **1_J2_**. The biphasic behaviour of the competing aggregation processes could be fitted using the cooperative growth model combined with a recently introduced competing dimerization model,^20^ giving an off-pathway dimerization constant of *K*_J1_ = 1.3 × 10^4^ M^–1^, a very small nucleation binding constant of *K*_N_ = 2.4 × 10^–2^ M^–1^, and an elongation binding constant of *K*_J2_ = 1.0 × 10^5^ M^–1^ (Fig. S5 in the ESI†). The Gibbs free energy Δ*G*° for each process was calculated by applying the values of *K*_J1_, *K*_N_, and *K*_J2_ to eqn (1), in which *R* is the ideal gas constant, *T* is the absolute temperature, and *K* is the equilibrium constant:

1 Δ*G*° = –*RT* ln *K*

**Fig. 5:**
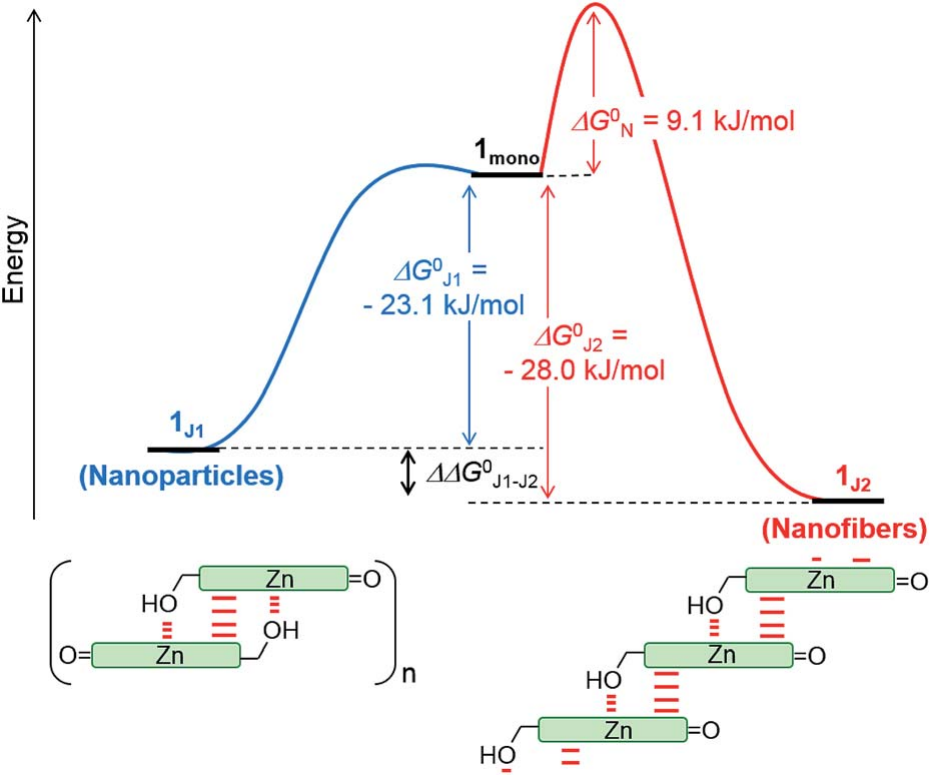
Schematic energy landscape of the interplay of two competing pathways for the formation of off-pathway nanoparticles composed of antiparallel dimers (**1_J1_**) and thermodynamically more stable helical nanofibers composed of parallel metallo-supramolecular π-stacks (**1_J2_**) of ZnChl **1** in a 40 : 60 MeOH/water mixture.

By merging the thermodynamic data we can derive an energy landscape, in which the formation of nuclei is the most energetically unfavourable step for the formation of thermodynamically stable **1_J2_** nanofibers (Fig. 5). It is important to point out that the values shown in Fig. 5 are obtained for a high methanol content (40%), *i.e.* a situation where the hydrophobic effect is still small. With a higher water content the nucleation barrier increases significantly and therefore **1_J1_** constitutes a deeply trapped state as illustrated by our kinetic data for the 1 : 99 MeOH/water mixture (Fig. 3b, green dots). This raises the question: how is the self-assembly of BChl dyes in natural chlorosomes controlled? Because living systems are in general not in thermodynamic equilibrium, it is anticipated that within chlorosomes out-of-equilibrium aggregate structures may exist. Subtle changes in environmental conditions would, however, foster nucleation processes, as similarly demonstrated in seeded supramolecular polymerizations,^16^ and initiate a change in the packing arrangement. Such structural changes may indeed be nature’s way of adapting chlorosomal light harvesting systems to different light intensities and of modulating functional properties such as exciton transport.9h,^21^ Accordingly, different aggregate morphologies for wild type and mutant strains of green bacteria might have their origin in the selection of different aggregation pathways. However, in *in vitro* experiments they may also arise by unintentional nucleation processes during sample preparation.

## Conclusions

In conclusion, we have investigated the self-assembly mechanism of a semi-synthetic model compound ZnChl dye **1**, which possesses high structural similarity to the natural BChl *c* scaffold. Our kinetic studies on self-assembly processes of ZnChl **1** in various MeOH/water solvent mixtures at different concentrations revealed the formation of nanoparticles with short-range J-coupling as an off-pathway intermediate with regard to the formation of thermodynamically more stable one-dimensional helical J-aggregate nanofibers. Based on a thermodynamic analysis that combines the cooperative *K*_2_–*K* growth model with a competing dimerization model, an energy landscape could be derived that describes the pathway complexity property of this system. Importantly, since the kinetic stability of the off-pathway nanoparticles increases with increasing concentration of ZnChl **1** or the water content in the MeOH/water solvent mixture, the off-pathway nanoparticle product gets trapped to such an extent that no transformation to the more ordered thermodynamic product is observed anymore within reasonable time scales for a water content >90%. Therefore, we assume that out-of-equilibrium aggregate structures of natural BChl dyes may also be present in natural chlorosomes. Furthermore, our results should raise awareness regarding the preparation conditions for metallochlorin dye aggregates, independently of whether they are of synthetic or natural origin. In particular, in an aqueous environment π-scaffolds are likely to self-assemble into kinetically trapped aggregate structures whose functional properties may be significantly different from those of the thermodynamically most stable aggregate structures.

## Conflicts of interest

There are no conflicts to declare.

## Supplementary Material

Supplementary information
