# Supplementary material for: Pathway complexity in the self-assembly of a zinc chlorin model system of natural bacteriochlorophyll J-aggregates
Source: Chem Sci. 2018 Feb 14;9(10):2768–73. doi: 10.1039/c7sc03725b (PMC5914135; doi:10.1039/c7sc03725b)
Supplement: Supplementary file 1 [file SC-009-C7SC03725B-s001.pdf]

Electronic Supplementary Material (ESI) for Chemical Science.
This journal is © The Royal Society of Chemistry 2018

Electronic Supplementary Information

Pathway complexity in self-assembly of a zinc chlorin model system of natural bacteriochlorophyll J-aggregates

Soichiro Ogi,[a,b,§] Charlotte Grzeszkiewicz[a] and Frank Würthner*[a,b]

[a] Universität Würzburg, Institut für Organische Chemie, Am Hubland, 97074 Würzburg, Germany. E-mail: *wuerthner@uni-wuerzburg.de*

[b] Universität Würzburg, Center for Nanosystems Chemistry (CNC) & Bavarian Polymer Institute (BPI), Theodor-Boveri-Weg, 97074 Würzurg, Germany

[§] Current address: Department of Chemistry, Graduate School of Science, Nagoya University, Furo, Chikusa, Nagoya 464-8602, Japan

KEYWORDS: Bacteriochlorophyll, J-aggregate, metallochlorin, supramolecular polymerization, self-assembly pathway complexity

Materials and methods

Zinc chlorin ZnChl **1** was synthesized according to previously reported literature procedure.[S1] Spectroscopic measurements were conducted under ambient conditions using dry solvents. UV/vis absorption spectra were recorded on a Varian Cary 100 Bio and Perkin–Elmer Lambda 35 spectrometer. CD spectra were recorded on a JASCO J815 spectrophotometer. Atomic force microscopy (AFM) measurements were performed at ambient conditions with a Bruker AXS MultiModeTM 8 SPM system and Solver Next system from NT-MDT in semicontact mode. Silicon cantilevers (OMCL-AC160TS, Olympus) with a resonance frequency of ~300 kHz and spring constant of ~42 Nm^{-1} were used. The rates summarized in Table S1 were calculated from the changes in the ε and g values observed in the first 0.5 h or 1 h of the time-dependent UV/vis absorption and CD spectroscopic measurements.

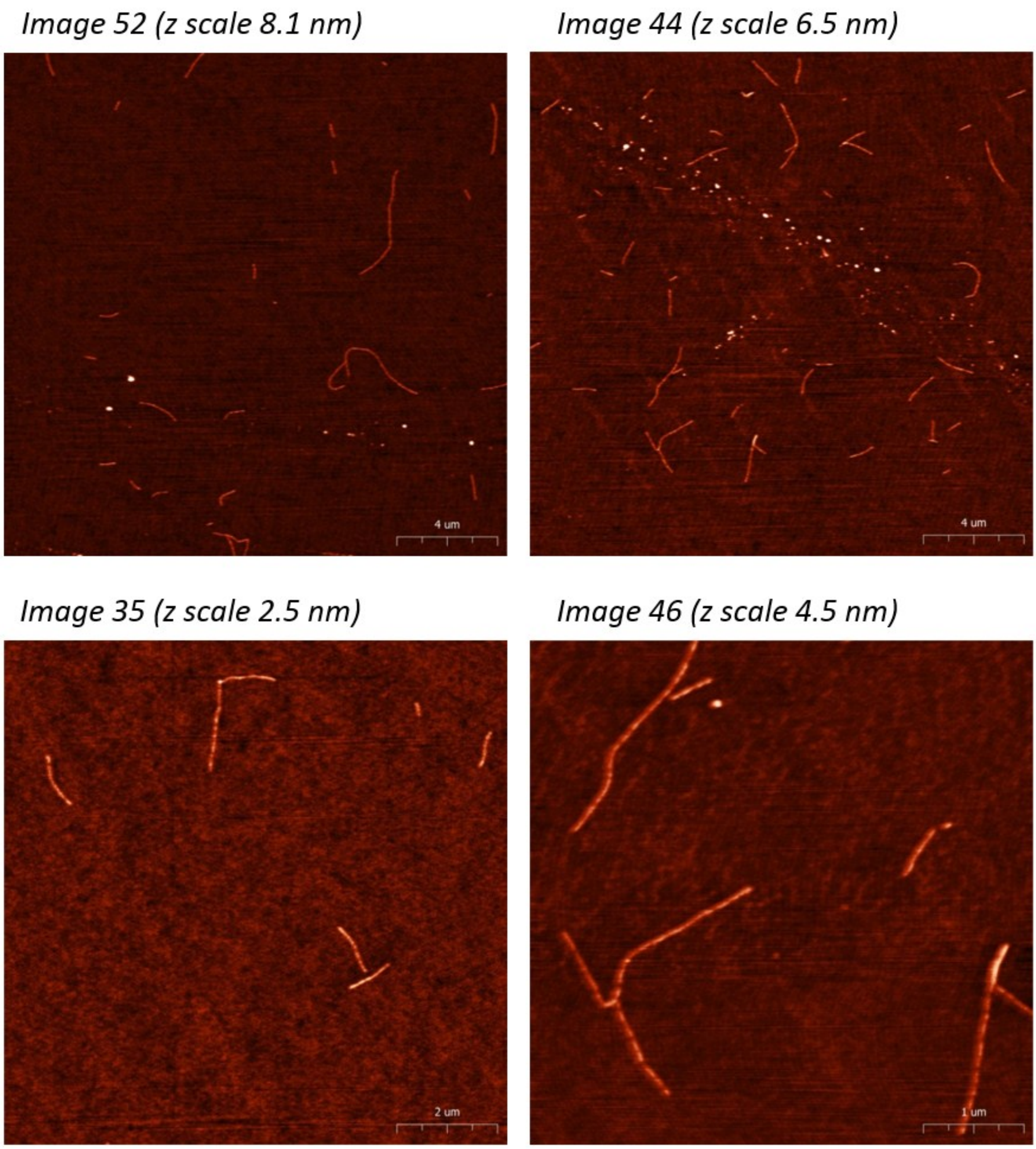

Fig. S1 Selection of AFM height images of $\mathbf{1}_{\mathbf{J2}}$ spin-coated onto silicon wafer from a freshly prepared 30:70 MeOH/water solution. For scale bars and z scale, see images.

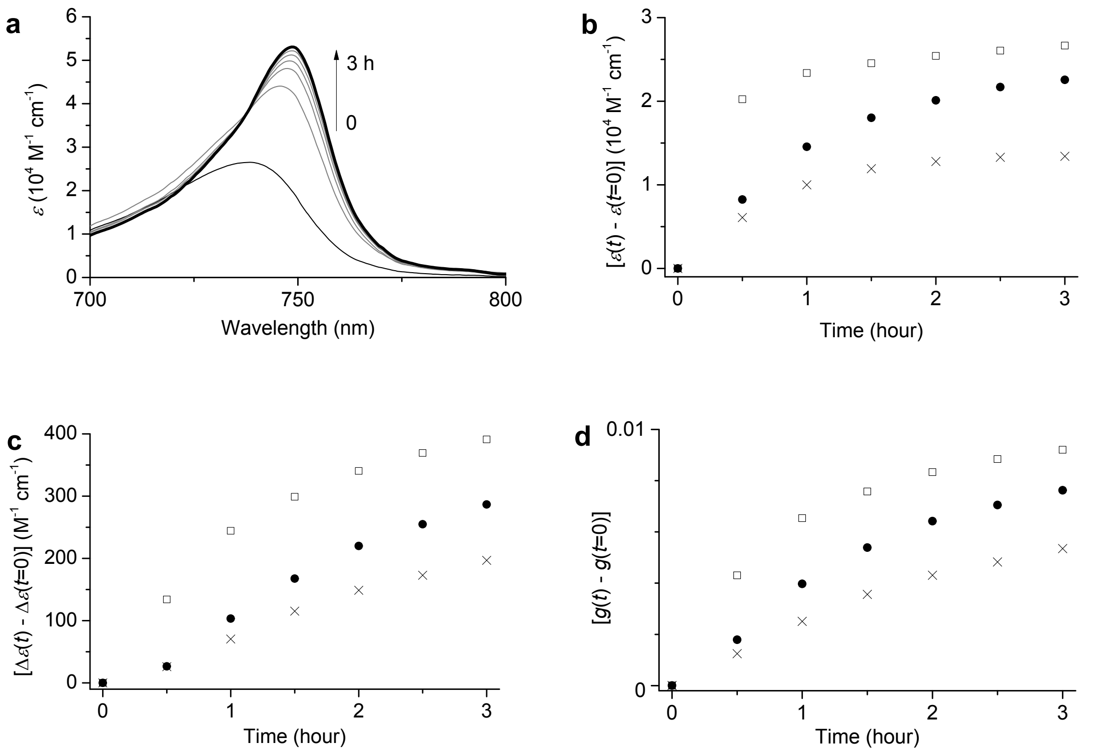

Fig. S2 (a) Time-dependent UV/vis absorption spectral changes showing the transformation from $\mathbf{1}_{\mathbf{J1}}$ to $\mathbf{1}_{\mathbf{J2}}$ in 30:70 MeOH/water solvent mixture at $c_T = 1 \times 10^{-5}$ M and 293 K. The arrows indicate spectral changes with increasing time from 0 to 3 h. (b-d) Time course of the transformation from $\mathbf{1}_{\mathbf{J1}}$ to $\mathbf{1}_{\mathbf{J2}}$ in 30:70 MeOH/water solvent mixture monitored by the time-dependent changes of (b) ε, (c) $\Delta\varepsilon$, and (d) g values at 745 nm at a temperature of 293 K and total concentrations of 1×10^{-5} (open squares), 2×10^{-5} (filled circles), and 3×10^{-5} M (crosses).

Table S1. Rates of the transformation from nanoparticles $\mathbf{1_{J1}}$ to one-dimensional fibril aggregates $\mathbf{1_{J2}}$ calculated from time-dependent changes of ε and g values[a)] at the given total concentrations (c_T) of ZnChl **1** in particular MeOH/water solvent mixture.

c_T (M)	MeOH/water (vol%)	$d\varepsilon/dt$ [b)] (M^{-1} cm^{-1} h^{-1})	$d(g)/dt$ [b)] (h^{-1})
1×10^{-5}	30:70	4.0×10^{4}	8.6×10^{-3}
2×10^{-5}	30:70	1.6×10^{4}	3.6×10^{-3}
3×10^{-5}	30:70	1.2×10^{4}	2.5×10^{-3}
1×10^{-5}	20:80	1.4×10^{4}	2.5×10^{-3}
1×10^{-5}	10:90	0.3×10^{3} [c)]	0.4×10^{-3} [c)]

[a)] Changes at the wavelength of 745 nm and at 293 K. [b)] Calculated from the ε and g values obtained in the first 0.5 (first three entries) or 1 h (fourth entry) of the time-dependent measurements. [c)] Calculated from time-dependent changes observed after a lag time of about 3 h.

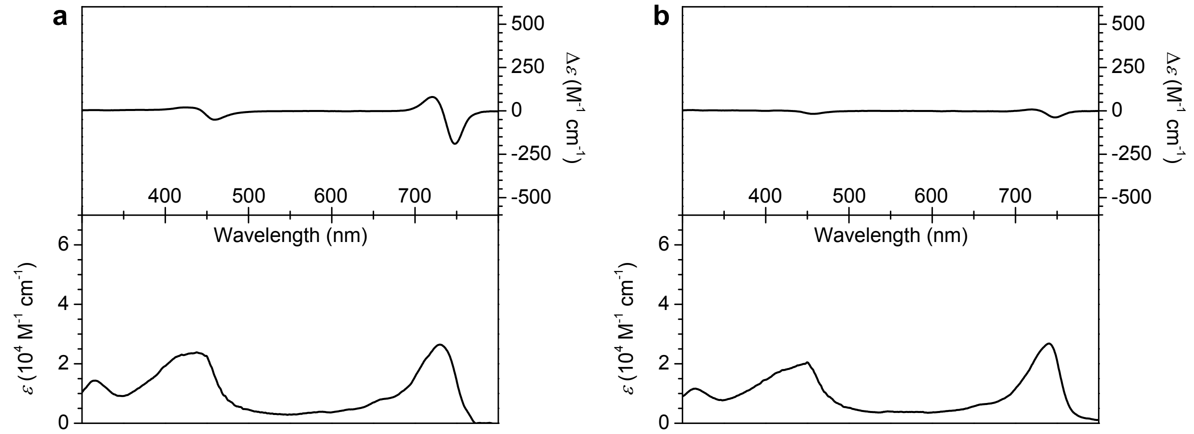

Fig. S3 Solvent-dependent CD and UV/vis absorption spectra of freshly prepared solutions of **1** in MeOH/water solvent mixtures with volume ratios of (a) 10:90, and (b) 1:99 at a concentration of 1×10^{-5} M and a temperature of 293 K.

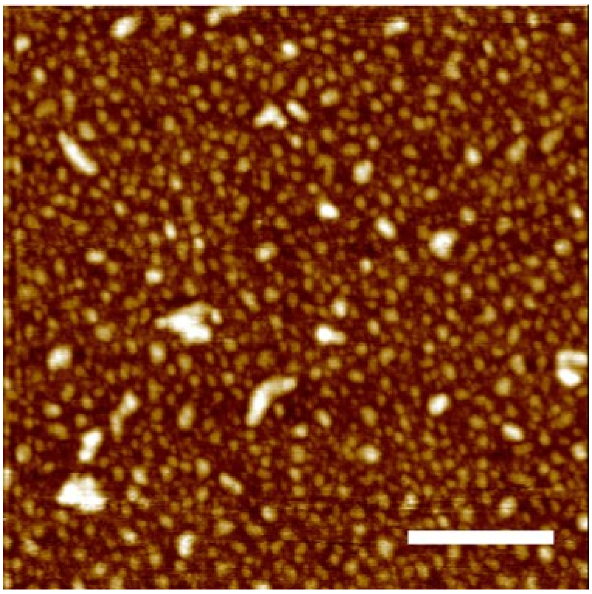

Fig. S4 AFM height image of $\mathbf{1}_{\mathbf{J1}}$ spin-coated onto a silicon wafer from a freshly prepared 1:99 MeOH/water solution. Scale bar, 250 nm.

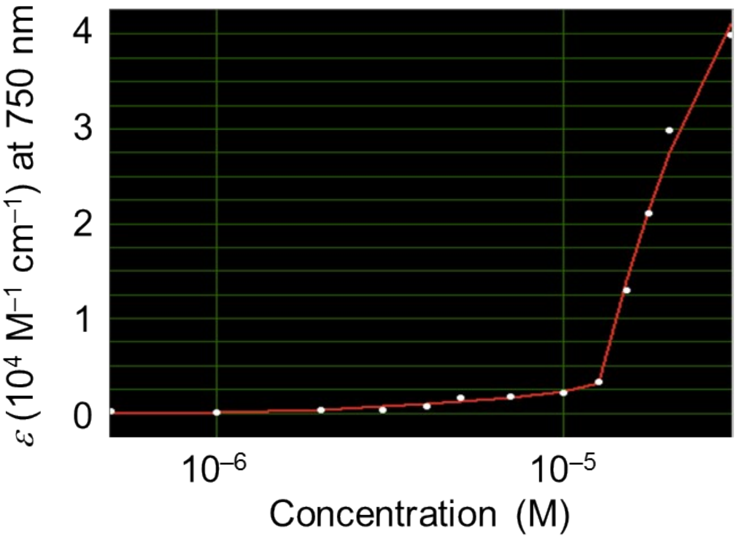

Fig. S5 Application of the K_2-K cooperative model with additional off-pathway dimer species to observed absorption changes at 750 nm. The red line shows the resulting fit of the two competitive aggregation processes calculated according to the cooperative growth model combined with competing dimerization model.[S2]

Reference

S1) V. Huber, S. Sengupta and F. Würthner, *Chem. Eur. J.*, 2008, **14**, 7791–7807.

S2) F. Fennel, S. Wolter, Z. Xie, P.-A. Plötz, O. Kühn, F. Würthner, and S. Lochbrunner, *J. Am. Chem. Soc.*, 2013, **135**, 18722−18725.
